# Endoplasmic Reticulum Stress Contributes to Gefitinib-Induced Apoptosis in Glioma

**DOI:** 10.3390/ijms22083934

**Published:** 2021-04-11

**Authors:** Cheng-Yi Chang, Ping-Ho Pan, Chih-Cheng Wu, Su-Lan Liao, Wen-Ying Chen, Yu-Hsiang Kuan, Wen-Yi Wang, Chun-Jung Chen

**Affiliations:** 1 Department of Surgery, Feng Yuan Hospital, Taichung 420, Taiwan; c.y.chang.ns@gmail.com; 2 Department of Pediatrics, Tungs’ Taichung MetroHarbor Hospital, Taichung 435, Taiwan; pph.pgi@gmail.com; 3 Department of Veterinary Medicine, National Chung Hsing University, Taichung 402, Taiwan; wychen@dragon.nchu.edu.tw; 4 Department of Anesthesiology, Taichung Veterans General Hospital, Taichung 407, Taiwan; chihcheng.wu@gmail.com; 5 Department of Medical Research, Taichung Veterans General Hospital, Taichung 407, Taiwan; slliao@vghtc.gov.tw; 6 Department of Pharmacology, Chung Shan Medical University, Taichung 402, Taiwan; kuanyh@csmu.edu.tw; 7 Department of Nursing, HungKuang University, Taichung 433, Taiwan; walice@sunrise.hk.edu.tw; 8 Department of Medical Laboratory Science and Biotechnology, China Medical University, Taichung 404, Taiwan

**Keywords:** apoptosis, EGFR inhibitors, ER stress, glioma, Noxa

## Abstract

Adequate stress on the Endoplasmic Reticulum (ER) with the Unfolded Protein Response (UPR) could maintain glioma malignancy. Uncontrolled ER stress, on the other hand, predisposes an apoptosis-dominant UPR program. We studied here the proapoptotic actions of the Epidermal Growth Factor Receptor (EGFR) inhibitor gefitinib, with the focus on ER stress. The study models were human H4 and U87 glioma cell lines. We found that the glioma cell-killing effects of gefitinib involved caspase 3 apoptotic cascades. Three branches of ER stress, namely Activating Transcription Factor-6 (ATF6), Protein Kinase R (PKR)-Like ER Kinase (PERK), and Inositol-Requiring Enzyme 1 (IRE1), were activated by gefitinib, along with the elevation of intracellular free Ca^2+^, Reactive Oxygen Species (ROS), and NADPH Oxidase2/4 (NOX2/4). Specifically, elevated IRE1 phosphorylation, Tumor Necrosis Factor (TNF) Receptor-Associated Factor-2 (TRAF2) expression, Apoptosis Signal-Regulating Kinase-1 (Ask1) phosphorylation, c-Jun N-Terminal Kinase (JNK) phosphorylation, and Noxa expression appeared in gefitinib-treated glioma cells. Genetic, pharmacological, and biochemical studies further indicated an active ROS/ER stress/Ask1/JNK/Noxa axis causing the glioma apoptosis induced by gefitinib. The findings suggest that ER-stress-based therapeutic targeting could be a promising option in EGFR inhibitor glioma therapy, and may ultimately achieve a better patient response.

## 1. Introduction

While cancer cells are notorious for their high proliferation and quick spread, they are surrounded by hostile microenvironments such as hypoxia, nutrient deficiency, lactic acidosis, and oxidative stress. Intrinsic events from oncogenic activation, aerobic glycolysis, and high protein demands would disrupt cellular proteostasis, leading to Endoplasmic Reticulum (ER) stress. To overcome such obstacles and turn them into survival momentum, cancer cells possess several adaptive mechanisms. One of them is the ER stress-derived Unfolded Protein Response (UPR), which is crucial for their adaptation and survival [1,2]. Conversely, in the case of unsuccessful UPR in resolving ER stress, a dominant UPR program commits the cells to death [3]. The phenomena highlight a dual role of UPR in the progression of the malignancy and provide a means for therapeutic treatment.

UPR is coordinated by three different signaling branches of transmembrane proteins resident on ER: Protein Kinase R (PKR)-Like ER Kinase (PERK), Inositol-Requiring Enzyme 1 (IRE1), and Activating Transcription Factor 6 (ATF6). Under a homeostatic ER, the lumenal domains of and PERK, IRE1, and ATF6 interact with ER abundant chaperone Glucose-Regulated Protein 78 (GRP78) and remain in an inactive state. Once misfolded proteins accumulate, GRP78 titrates misfolded proteins, liberating PERK, IRE1, ATF6. The liberated PERK initiates autophosphorylation and phosphorylates Eukaryotic Translation Initiation Factor-2α (eIF2α), leading to a greater expression of ATF4 and C/EBP Homologous Protein (CHOP). Activated IRE1, which possesses kinase and RNase activities, yields a spliced form of transcription factor X-Box Binding Protein-1 (XBP1) mRNA. IRE1 also recruits Tumor Necrosis Factor (TNF) Receptor-Associated Factor-2 (TRAF2) to activate the Apoptosis Signal-Regulating Kinase-1 (Ask1)/c-Jun N-Terminal Kinase (JNK) axis. When released from GRP78, ATF6 enters the Golgi apparatus, generating a cleaved fragment that is a transcription factor. Transcription factors ATF4, CHOP, XBP1, and ATF6 have shared and individual programs, governing the expression of a variety of genes involved in cell survival, angiogenesis, metastasis, immune surveillance, inflammation, autophagy, and apoptosis [4]. Regarding malignancy, ER stress and three sensory branches are promising targets for intervention.

In the central nerve system, the most aggressive malignancy is glioma, particularly the glioblastoma multiforme. Despite conventional and novel treatment strategies, patients with malignant glioma have a poor prognosis, with high recurrence rates [5]. Genetic mutation and amplification of the Epidermal Growth Factor Receptor (EGFR) in glioma highlight an opportunity to treat patients using EGFR tyrosine kinase inhibitors. Although EGFR inhibitors have promising potential, clinical application remains challenging with monotherapy or combination therapy in combating malignant glioma. Clinical findings reveal that only 10–20% of malignant glioma patients benefit from the gefitinib and the coexpression of the EGFRvIII oncogene, and the PTEN tumor suppressor protein in patients favors a clinical response to EGFR inhibitor therapy. Besides genetic status, the combination therapy with gefitinib and temozolomide represents an alternative option [6,7,8,9,10,11]. The phenomena underscore the importance of a better understanding of anti-neoplastic mechanisms caused by EGFR inhibitors such as gefitinib.

Glioma cells over-express markers of UPR, which contribute to proliferation, angiogenesis, resistance, and stem cell-like activity, and are correlated with a poor prognosis [12,13,14]. However, anti-glioma treatments cause ER stress and UPR. With extrinsic insults and intrinsic alterations, glioma cells become sensitized to ER stress, leading to apoptosis [15,16,17,18,19]. The lung cancer cells with EGFR inhibitor gefitinib-resistance express higher levels of GRP78, and lower levels of CHOP. The induction of ER stress sensitizes these cancer cells to gefitinib therapy [20,21]. Currently, the alteration and role of ER stress in gefitinib-treated glioma cells remain unclear. In our previous studies on glioma cells, we reported that gefitinib induces apoptosis and autophagy, and the ER stress contributes to glioma apoptosis [15,16,22,23,24]. To extend the finding on ER stress, we conducted this study to determine the role of ER stress in gefitinib-induced glioma apoptosis, and identify the molecular basis underlying the UPR-committed apoptotic program.

## 2. Results

### 2.1. Gefitinib Induced ER Stress in H4 Cells

**Figure 1:**
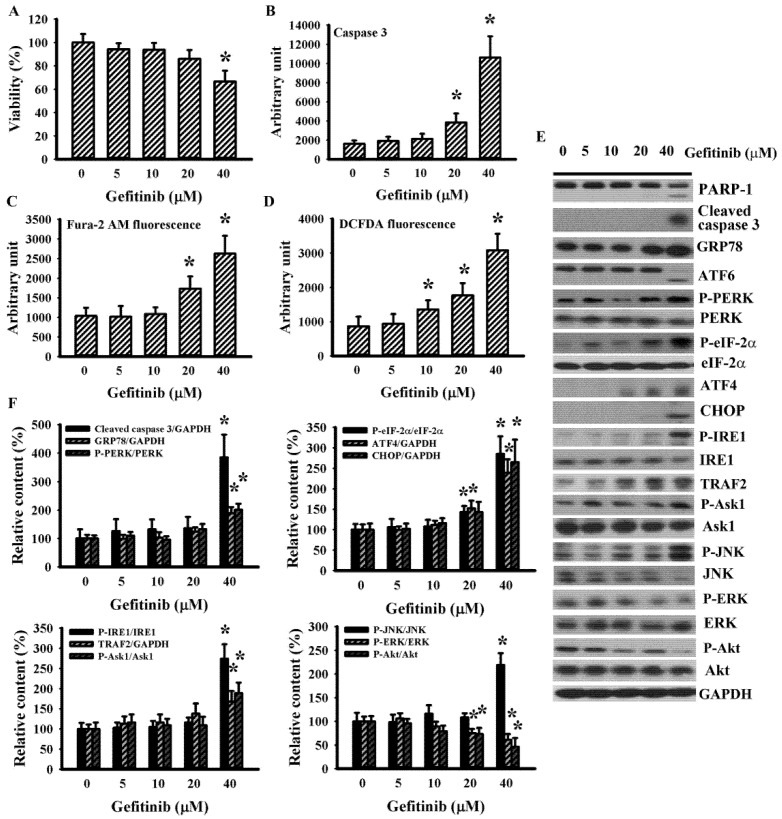
Gefitinib caused apoptosis and Endoplasmic Reticulum (ER) stress in H4 cells. H4 cells were treated with various concentrations of gefitinib (0–40 μM). Cell viability (24 h) was measured with the alamarBlue assay (**A**). Caspase 3 activity (6 h) was measured with enzymatic assay (**B**). Intracellular free Ca^2+^ (6 h) was measured with Fura-2 AM fluorescence (**C**). Reactive Oxygen Species (ROS; 6 h) was measured with DCFDA fluorescence (**D**). Protein extracts (6 h) were subjected to Western blot with indicated antibodies. Representative blots (**E**) and quantitative data (**F**) of three independent experiments are shown. Protein contents were normalized with corresponding total protein or GAPDH. * *p* < 0.05 vs. untreated control, *n* = 4 (**A**–**D**).

**Figure 2:**
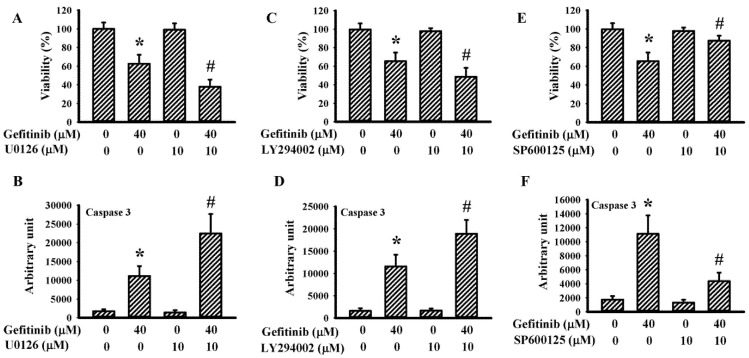
Pharmacological inhibitors altered gefitinib-induced apoptosis in H4 cells. H4 cells were treated with gefitinib (0 and 40 μM) together, with or without U0126 (10 μM, **A**,**B**), LY294002 (10 μM, **C**,**D**), or SP600125 (10 μM, **E**,**F**). Cell viability (24 h) was measured with the alamarBlue assay (**A**,**C**,**E**). Caspase 3 activity (6 h) was measured with enzymatic assay (**B**,**D**,**F**). * *p* < 0.05 vs. untreated control and # *p* < 0.05 vs. gefitinib (40 μM), *n* = 4.

To explore the potential involvement of ER stress in gefitinib-induced glioma cell death in H4 cells, parameters of UPR and viability-associated molecules were measured. With higher concentrations, gefitinib produced a host of changes: viability loss (Figure 1A); caspase 3 activation (Figure 1B); intracellular mobilization of free Ca^2+^ (Figure 1C); Reactive Oxygen Species (ROS) generation (Figure 1D); protein levels’ upregulation in cleaved caspase 3, GRP78, ATF4, CHOP, and TRAF2; protein hyperphosphorylation in PERK, eIF2α, IRE1, Ask1, and JNK; protein hypo-phosphorylation in Extracellular Signal-Regulated Kinase (ERK), and Akt; and Poly(ADP-ribose) Polymerase-1 (PARP-1), and ATF6 proteolytic cleavage (Figure 1E,F). ERK inhibitor U0126 (Figure 2A,B) and PI3K/Akt inhibitor LY294002 (Figure 2C,D) augmented gefitinib-induced viability loss (Figure 2A,C) and caspase 3 activation (Figure 2B,D), while JNK inhibitor SP600125 alleviated gefitinib-induced viability loss (Figure 2E) and caspase 3 activation (Figure 2F). Findings were consistent with substantial roles of ERK, JNK, and PI3K/Akt, signaling axes in gefitinib-induced apoptosis of glioma.

### 2.2. 4-Phenylbutyrate and IRE1 Silencing Alleviated Gefitinib-Induced Glioma Apoptosis in H4 Cells

**Figure 3:**
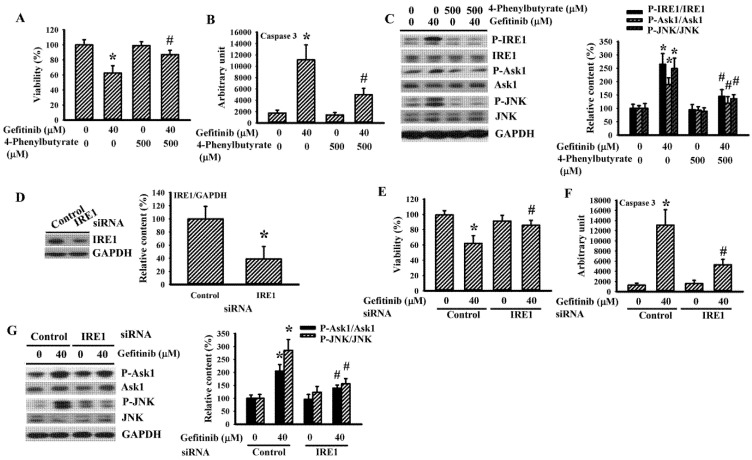
4-Phenylbutyrate and Inositol-Requiring Enzyme 1 (IRE1) silencing alleviated gefitinib-induced apoptosis in H4 cells. H4 cells were treated with gefitinib (0 and 40 μM) together, with or without 4-phenylbutyrate (500 μM, **A**–**C**). Cell viability (24 h) was measured with the alamarBlue assay (**A**). Caspase 3 activity (6 h) was measured with enzymatic assay (**B**). Protein extracts (6 h) were subjected to Western blot with indicated antibodies. Representative blots of three independent experiments and quantitative data are shown (**C**). H4 cells were transfected with control siRNA (1 nM) or IRE1 siRNA (1 nM) for 24 h. Protein extracts were subjected to Western blot with indicated antibodies. Representative blots of three independent experiments and quantitative data are shown (**D**). The transfected cells were then treated with gefitinib (0 and 40 μM). Cell viability (24 h) was measured with the alamarBlue assay (**E**). Caspase 3 activity (6 h) was measured with enzymatic assay (**F**). Protein extracts (6 h) were subjected to Western blot with indicated antibodies. Representative blots of three independent experiments and quantitative data are shown (**G**). Protein contents were normalized with corresponding total protein or GAPDH. * *p* < 0.05 vs. untreated control and # *p* < 0.05 vs. gefitinib (40 μM), *n* = 4 (**A**,**B**,**E**,**F**).

The chemical molecular chaperone 4-phenylbutyrate is known to restore ER homeostasis through IRE1 [25]. It also alleviated gefitinib-induced H4 cell viability loss (Figure 3A), caspase 3 activation (Figure 3B) and protein hyperphosphorylation of IRE1, Ask1, and JNK (Figure 3C). RNA interference was achieved by delivering siRNA, causing a reduced level of endogenous IRE1 protein (Figure 3D). IRE1-silenced H4 cells were refractory to gefitinib-induced viability loss (Figure 3E), caspase 3 activation (Figure 3F), and protein hyperphosphorylation of Ask1 and JNK (Figure 3G), compared with control cells. Findings were consistent with a role of the 4-phenylbutyrate-inhibitable IRE1 axis in the gefitinib-induced apoptosis of glioma.

### 2.3. BAPTA-AM and N-Acetyl-Cysteine (NAC) Alleviated Gefitinib-Induced Glioma Apoptosis in H4 Cells

**Figure 4:**
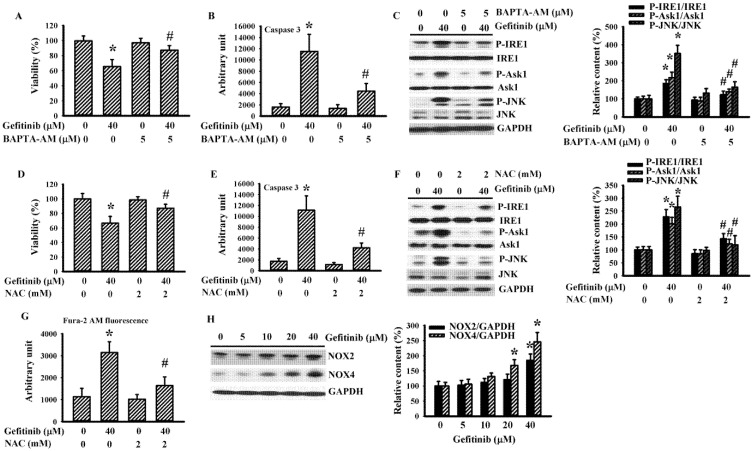
BAPTA-AM and NAC alleviated gefitinib-induced apoptosis in H4 cells. H4 cells were treated with gefitinib (0 and 40 μM) together, with or without BAPTA-AM (5 μM, **A**–**C**), or NAC (2 mM, **D**–**G**). Cell viability (24 h) was measured with the alamarBlue assay (**A**,**D**). Caspase 3 activity (6 h) was measured with enzymatic assay (**B**,**E**). Protein extracts (6 h) were subjected to Western blot with indicated antibodies. Representative blots of three independent experiments and quantitative data are shown (**C**,**F**). Intracellular free Ca^2+^ (6 h) was measured with Fura-2 AM fluorescence (**G**). H4 cells were treated with various concentrations of gefitinib (0–40 μM). Protein extracts (6 h) were subjected to Western blot with indicated antibodies. Representative blots of three independent experiments and quantitative data are shown (**H**). Protein contents were normalized with corresponding total protein or GAPDH. * *p* < 0.05 vs. untreated control and # *p* < 0.05 vs. gefitinib (40 μM), *n* = 4 (**A**,**B**,**D**,**E**,**G**).

Gefitinib increased intracellular levels of free Ca^2+^ and induced ROS generation in H4 cells (Figure 1C,D). To further study their roles, we applied intracellular calcium chelating agent BAPTA-AM and antioxidant NAC to these cells. BAPTA-AM (Figure 4A–C) and NAC (Figure 4D–F) alleviated gefitinib-induced cell viability loss (Figure 4A,D), caspase 3 activation (Figure 4B,E) and protein hyperphosphorylation of IRE1, Ask1, and JNK (Figure 4C,F). NAC also alleviated gefitinib-induced intracellular free Ca^2+^ (Figure 4G). Gefitinib had increased NADPH Oxidase-2 (NOX2) and NOX4 protein expression in H4 cells (Figure 4H). These NOX family members are likely involved in IRE1-mediated ER stress [26]. Our findings suggest substantial roles played by free Ca^2+^ and ROS in the gefitinib-induced ER stress and apoptosis, with NOX2/4 as candidate sources for ROS generation.

### 2.4. Gefitinib Induced ER Stress and Apoptosis in U87 Cells

**Figure 5:**
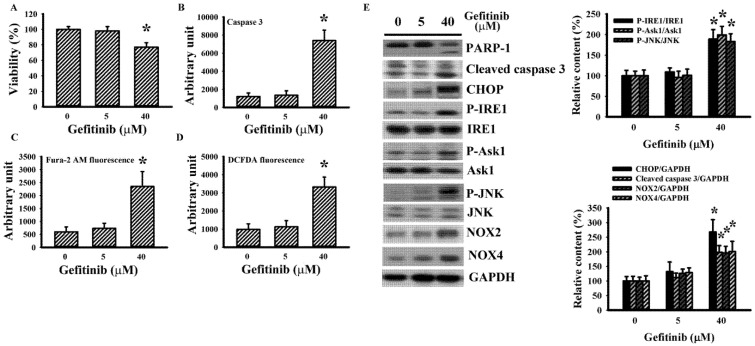
Gefitinib caused apoptosis and ER stress in U87 cells. U87 cells were treated with various concentrations of gefitinib (0–40 μM). Cell viability (24 h) was measured with the alamarBlue assay (**A**). Caspase 3 activity (6 h) was measured with enzymatic assay (**B**). Intracellular free Ca^2+^ (6 h) was measured with Fura-2 AM fluorescence (**C**). ROS (6 h) was measured with DCFDA fluorescence (**D**). Protein extracts (6 h) were subjected to Western blot with indicated antibodies. Representative blots of three independent experiments and quantitative data are shown (**E**). Protein contents were normalized with corresponding total protein or GAPDH. * *p* < 0.05 vs. untreated control, *n* = 4 (**A**–**D**).

U87 cells were also studied, similar to H4 cells. Gefitinib caused ER stress and apoptosis in U87 cells by triggering viability loss (Figure 5A); caspase 3 activation (Figure 5B); intracellular free Ca^2+^ mobilization (Figure 5C); ROS generation (Figure 5D); proteolytic cleavage of PARP-1; protein hyperphosphorylation of IRE1, Ask1, and JNK; and protein upregulation in cleaved caspase 3, CHOP, NOX2 and NOX4 (Figure 5E). That is, gefitinib has a pro-apoptotic potential against glioma cells involving ER stress.

### 2.5. Gefitinib Caused Noxa Upregulation in Glioma Cells

**Figure 6:**
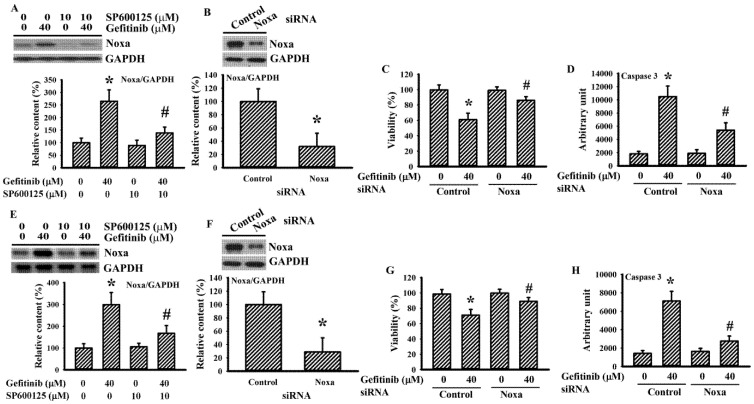
Noxa is crucial to gefitinib-induced apoptosis in glioma cells. H4 (**A**) and U87 (**E**) cells were treated with gefitinib (0 and 40 μM) together, with or without SP600125 (10 μM). Protein extracts (6 h) were subjected to Western blot with indicated antibodies. Representative blots of three independent experiments and quantitative data are shown. H4 (**B**–**D**) and U87 (**F**–**H**) cells were transfected with control siRNA (1 nM) or Noxa siRNA (1 nM) for 24 h. Protein extracts were subjected to Western blot with indicated antibodies. Representative blots of three independent experiments and quantitative data are shown (**B**,**F**). The transfected cells were then treated with gefitinib (0 and 40 μM). Cell viability (24 h) was measured with the alamarBlue assay (**C**,**G**). Caspase 3 activity (6 h) was measured with enzymatic assay (**D**,**H**). Protein contents were normalized with GAPDH. * *p* < 0.05 vs. untreated control and # *p* < 0.05 vs. gefitinib (40 μM), *n* = 4 (**C**,**D**,**G**,**H**).

BH3-only proteins are candidate downstream effectors in linking ROS/ER stress and apoptosis [27]. Gefitinib caused an elevated expression of the Noxa protein in H4 cells and the elevation was alleviated by SP600125 (Figure 6A). Silence of endogenous Noxa (Figure 6B) protected H4 cells against gefitinib-induced cell viability loss (Figure 6C) and caspase 3 activation (Figure 6D). The above-mentioned findings were duplicated in U87 cells (Figure 6E–H). Findings were consistent with ROS/ER stress/Ask1/JNK/Noxa axis being an apoptotic cause in gefitinib-treated glioma cells.

## 3. Discussion

**Figure 7:**
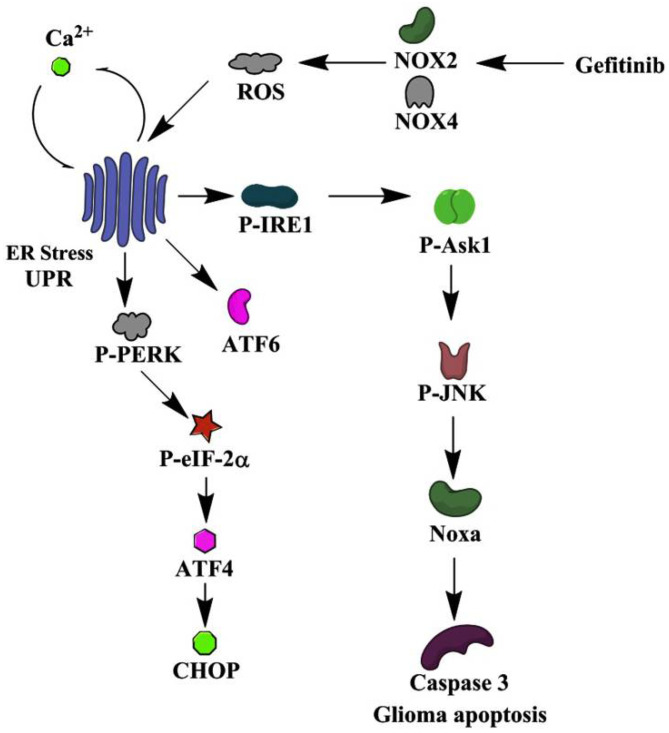
A possible schema of apoptotic mechanism in glioma cells elicited by gefitinib is proposed. The schematic diagram indicates the cellular molecules, signaling molecules, and cascades used in mediating the induction of ER stress/Unfolded Protein Response (UPR) and the consequences of apoptosis after gefitinib treatment in glioma cells. Some additional signaling molecules and cascades were omitted for the sake of clarity.

Over-expressions of GRP78 and UPR components have been implicated in malignant glioma of aggressive phenotypes, while ER stress also predisposes glioma cells to apoptosis upon therapeutic treatments [10,11,12,13,14,15,16,17,19]. Although ER stress sensitizes lung cancer cells to gefitinib therapy [20,21], the involvement of ER stress in the actions of gefitinib on glioma cells is not clear. Continuing our earlier study on gefitinib-mediated glioma apoptosis [22], we had found here that gefitinib-induced glioma apoptosis was parallel with the following events: elevated GRP78, ATF4, and CHOP protein expressions; PERK, eIF2α, and IRE1 protein phosphorylation; ATF6 proteolytic cleavage; free Ca^2+^ mobilization; and ROS generation, crucial signs of ER stress. Pharmacological and genetic studies further identified an ROS/ER stress/Ask1/JNK/Noxa axis involving NOX2/4 in mediating gefitinib-induced glioma apoptosis (Figure 7). Besides EGF/EGFR signaling, current findings are consistent with the ROS/ER stress axis likely being a target for the anti-glioma actions of gefitinib.

Components of the UPR own both shared and individual biological activities. Glioma strongly expresses PERK, ATF4, ATF6, IRE1, and XBP1, and is associated with the malignant phenotype and poor prognosis [12,14,28]. Furthermore, IRE1 is also implicated in the expression of EGFR ligand epiregulin [29]. The knockdown of PERK, ATF6, or IRE1 reduces glioma cell viability with a greater sensitivity to stress-induced cell death [30,31,32]. Conversely, ER stress also leads to glioma cell death [15,16,17,18,19,20]. In an earlier study, we demonstrated that aspirin and indomethacin increased UPR component protein expression, and protein phosphorylation, resulting in glioma apoptosis. ER stress inhibitors such as salubrinal and 4-phenylbutyrate, calcium chelator BAPTA-AM, and antioxidant PDTC alleviate glioma apoptosis [15,16]. Upon gefitinib treatment, glioma cells elevated UPR components in protein expression and protein phosphorylation, along with apoptosis. Gefitinib-induced glioma apoptosis was suppressed by 4-Phenylbutyrate, BAPTA-AM, NAC, IRE1 silencing, and Noxa silencing. Although the fine-tuning of the UPR in directing glioma cells to survival or death is not fully understood, our current findings have suggested that therapeutic or cancer cell-killing agents commit UPR to the pro-death program.

Regarding the apoptotic program, Bcl-2 family members are transcriptional targets of ATF4, CHOP, XBP1, and ATF6 [4]. Other evidence indicated that IRE1/TRAF2/Ask1/JNK, p38 represents an alternative mechanism to initiate apoptosis by phosphorylating and inhibiting anti-apoptotic activity of Bcl-2 members, or activating pro-apoptotic function of BH3-only proteins [33,34]. In indomethacin-treated glioma cells, activated ER stress/Ask1/p38 axis causes Akt inactivation and Mcl-1/FLIP downregulation, resulting in cell apoptosis [15]. Aspirin causes ER stress and Noxa-mediated glioma apoptosis and the death program is alleviated by silencing PERK or eIF2α [16]. Besides apoptotic induction, autophagic proteins synthesis and autophagic complexes assembly are also targets of ER stress. Gefitinib induces autophagic cell death in glioma cells involving ROS generation, and valproic acid augments ROS generation and autophagy in gefitinib-treated glioma cells [23,24]. Herein, we demonstrated an ROS/ER stress/IRE1/Ask1/JNK/Noxa axis being actively involved in the gefitinib-induced glioma apoptosis. Since JNK and Noxa have a dominant role in glioma apoptosis [35,36], the current findings further highlight a pharmacological target of JNK/Noxa in glioma therapeutic development.

ER-associated NOXs is one means of turning on the IRE1/JNK/Noxa axis through ROS generation [26,27]. Gefitinib increased NOX2/4 protein expression and ROS generation in glioma cells. Their ER stress caused by gefitinib were alleviated by NAC. Oxidative stress is typically paralleled by EGFR phosphorylation, and through the EGFR signaling, protecting against oxidative stress-induced apoptosis. Erlotinib, by inhibiting EGFR, induces metabolic oxidative stress through NOX4 in human head and neck cancer cells [37]. NOXs are overexpressed in glioma cells and support aerobic glycolysis and malignancy [38]. Although there is no direct evidence, our findings suggest that gefitinib interrupting the compensatory interplay between NOXs and EGFR signaling could lead to an overwhelmed NOX2/4 activation and ROS generation. The consequence is convergence of signals to an apoptosis dominant UPR program.

UPR components and biological consequences could vary. Their exact relationship highly and dynamically depends on the microenvironment. Apart from IRE1 and JNK, the precise involvement of NOXs, other UPR components, downstream effectors, and transcriptional events remain to be explored. Other than gefitinib, the effects of clinical relevant EGFR inhibitors centered on glioma apoptosis are of interest. Additionally, despite the encouraging apoptotic and autophagic consequences in cell studies [22,23,24], EGFR inhibitors, including gefitinib, have only marginal benefits in patients with recurrent malignant glioma. The clinical response to EGFR inhibitor monotherapy is limited to a certain genetic status such as *EGFRvIII*, *PTEN*, and *IDH1*. To improve clinical response, combination therapy could be an option [6,7,8,9,10,11]. These to-be-solved issues should be addressed in future studies.

In conclusion, we found that gefitinib exhibited anti-neoplastic effects against glioma H4 and U87 cells through apoptosis, involving the ROS/ER stress/Ask1/JNK/Noxa axis. Doses of gefitinib (~40 μM) inducing apoptotic cell death in glioma cells are way higher than those inhibiting EGFR signaling (~5 μM). Once gefitinib treatment is effective, ER stress overrides survival machinery, predisposing cells to the apoptosis dominant program. Therefore, ER stress might well be valuable targets in combination therapy using gefitinib to combat malignant glioma.

## 4. Materials and Methods

### 4.1. Cell Cultures

Human U87 glioblastoma cell line (ATCC HTB-14) and H4 neuroglioma cell line (ATCC HTB-148) purchased from American Type Culture Collection (ATCC, Manassas, VA, USA) were maintained in Dulbecco’s modified Eagle medium (DMEM), containing 10% fetal bovine serum (FBS) for propagation. All experiments were conducted on cells placed in DMEM containing 2% FBS. H4 cells harbor genetic mutations in *PTEN* and one copy loss in *TP53*, but are not EGFRvIII variant. U87 cells are *PTEN*-deficient and *TP53*- and *EGFR*-wild type. Both cells are *Isocitrate Dehydrogenase (IDH)*-wild type [22].

### 4.2. Cell Viability Assay

Cells were plated onto 96-well plates 24 h prior to treatment. Cell viability was determined with an assay kit (alamarBlue™ Cell Viability Reagent) (ThermoFisher Scientific, Waltham, MA, USA). The optical absorbance at 570 and 600 nm was measured with a spectrophotometer.

### 4.3. Caspase 3 Activity Assay

Cells were plated onto 6-well plates 24 h prior to treatment. Caspase 3 activity was measured within isolated protein extracts (10 μg) according to instructions of the Caspase Fluorometric Assay kit (BioVision, Mountain View, CA, USA). Fluorescent signals of released AMC moiety were measured with a fluorometer (E_x_ 380 nm and E_m_ 460 nm).

### 4.4. Measurement of ROS

Cells were plated onto 96-well plates 24 h prior to treatment. To detect ROS, cells were loaded with 2′,7′-Dichlorofluorescein Diacetate (DCFDA, 5 μM; Molecular Probes, Eugene, OR, USA) for 30 min. Fluorescent signals were then measured in a fluorometer with excitation/emission at 495 nm/529 nm.

### 4.5. Measurement of Cytosolic Ca^2+^

Cells were plated onto 96-well plates 24 h prior to treatment. To detect intracellular free Ca^2+^, cells were loaded with Fura-2-Acetoxymethyl Ester (Fura-2 AM, 4 μM; Molecular Probes, Eugene, OR, USA). Fluorescent signals were measured in a fluorometer with dual excitation at 340 nm and 380 nm, with emission detected at 510 nm.

### 4.6. Western Blot

Cells were plated onto 6-well plates 24 h prior to treatment. At the end of treatments, cells were homogenized in the Laemmli SDS buffer. Equal amounts of protein extracts were separated and analyzed in a standard SDS-PAGE, followed by reacting to horseradish peroxidase-labeled IgG, Enhanced Chemiluminescence (ECL) visualization, and densitometric measurement. Primary antibodies were used to recognize the following proteins: PARP-1 (1:1000), cleaved caspase 3 (1:1000), GRP78 (1:1000), ATF6 (1:1000), PERK (1:1000), phospho-PERK (1:500, Thr-981), eIF-2α (1:1000), phospho-eIF2α (1:500, Ser-52), ATF4 (1:1000), CHOP (1:2000), IRE1 (1:1000), phospho-IRE1 (1:500, Ser-724), TRAF2 (1:1000), Ask1 (1:1000), phospho-Ask1 (1:500, Thr-845), JNK (1:1000), phospho-JNK (1:500, Thr-183/Tyr-185), ERK (1:1000), phospho-ERK (1:500, Thr-202/Tyr-204), Akt (1:1000), phospho-Akt (1:500, Ser-473), NOX2 (1;1000), NOX4 (1:1000), Noxa (1:1000; Santa Cruz Biotechnology, Santa Cruz, CA, USA), and Glyceraldehyde-3-Phosphate Dehydrogenase (GAPDH, 1:3000; R&D Systems, Minneapolis, MN, USA).

### 4.7. Small Interfering RNA (siRNA) Transfection

The siRNA against human IRE1 (sc-40705), Noxa (sc-37305), and control siRNA (sc-37007; Santa Cruz Biotechnology, Santa Cruz, CA, USA) were delivered to H4 cells using the INTERPERin siRNA transfection reagent (Polyplus-transfection, New York, NY, USA).

### 4.8. Statistical Analyses

Data were represented as mean ± standard deviation, and depicted with Sigma Plot 12.3 software. Statistical comparisons were analyzed using one-way or two-way analysis of variance, followed by Tukey or Dunnett post hoc test. Statistical significance was set at *p* < 0.05.
